# Is vision in schizophrenia characterized by a generalized reduction?

**DOI:** 10.3389/fpsyg.2013.00999

**Published:** 2013-12-27

**Authors:** Bernt C. Skottun, John R. Skoyles

**Affiliations:** ^1^Independent ScholarOslo, Norway; ^2^Centre for Mathematics and Physics in the Life Sciences and Experimental Biology, University College LondonLondon, UK

**Keywords:** schizophrenia, vision, sensitivity, VEPs, magnocellular

How the visual capabilities of those with schizophrenia differ from those of individuals without schizophrenia is a topic of active research. Of special interest is the question of whether or not they might have a magnocellular deficiency. It has been concluded that contrast sensitivity in schizophrenic subjects is characterized by a general reduction in sensitivity, and so does not indicate a magnocellular deficiency (Skottun and Skoyles, 2007). Likewise, many of the reported cases of abnormal visual masking linked to schizophrenia can be described by a general reduction (see, e.g., Rassovsky et al., 2004). Also this is hard to reconcile with a magnocellular deficit since, according to the theory, such a deficit would have been expected to cause a reduction that was related specifically to the U-shaped Type-B masking function (Skottun and Skoyles, 2009). These observations prompt the question of whether or not other differences between schizophrenic subjects and controls that have been attributed to magnocellular deficiencies can also be accounted by a general reduction in sensitivity or response.

Differences between schizophrenic subjects and nonschizophrenic controls attributed to magnocellular deficiencies have been found using visually evoked potentials (VEP). Butler et al. (2009) obtained VEP data for schizophrenic subjects and controls under two conditions. One condition aimed at predominantly stimulating the magnocellular system, the other the parvocellular system. Butler et al. (2009) found statistically significant reduction in the responses from the schizophrenic subjects, relative to those of controls, under the condition favoring the magnocellular system but not under the parvocellular condition. The authors interpreted this as evidence for a magnocellular deficiency linked to schizophrenia. The present report examines the possibility that these results could reflect instead a general response reduction.

The data of Butler et al. (2009) have been re-plotted in Figure 1. The open and filled symbols give the results for the schizophrenic subjects and controls, respectively, while panels A and B give the data obtained under the magno- and parvocellular conditions. A general response reduction was modeled by a simple linear scaling of the response from the control group. The scaling factor was determined by the best fit to the data of the schizophrenic subjects by calculating the smallest sum of squared deviations. Analyses of both magno- and parvocellular data conditions were included, giving a single scaling value of 0.756. (The scaling factors for the magno- and parvocellular data sets computed separately were 0.737 and 0.797, respectively). The scaled data are indicated by the dashed lines in Figure 1. Even though the data for the schizophrenic subjects in the magnocellular condition in some instances are slightly below the dashed line and the data in the parvocellular condition in some cases are slightly above the dashed line, the overall finding is that the scaled data give close fits to the data for the schizophrenic subjects for both conditions. This suggests that Butler et al.'s (2009) data for schizophrenic subjects in both the magnocellular and the parvocellular conditions are consistent with scaling by the same factor. This is consistent with a general reduction in the response rather than a difference (as suggested by Butler et al., 2009) between the magnocellular and parvocellular systems. No reason therefore exists to account for these data with a deficiency linked specifically to the magnocellular system.

**Figure 1:**
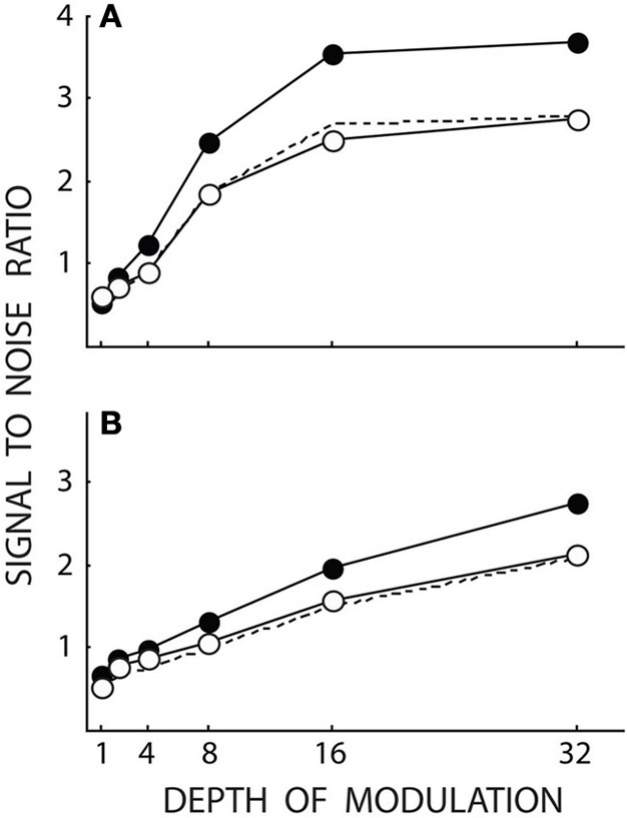
**Data re-plotted from Figure 4 of Butler et al. (2009).** Open and filled symbols give data for schizophrenic subjects (*N* = 20) and controls (*N* = 17), respectively. **(A,B)** Give results obtained under conditions aimed at stimulating predominately the magno- and parvocellular systems, respectively. The dashed lines give the results obtained by simply scaling the results for the controls in each panel by 0.756. As can be seen from the dashed lines, the data for schizophrenic subjects are close to the prediction based on general scaling.

One explanation for the differences between controls and schizophrenic subjects being significant in the magnocellular condition but not in the parvocellular one could be that the response differences were larger in the magnocellular condition. The fact that the responses were larger in this condition would have made the difference between the groups (as a result of scaling by a constant) larger under this condition, and so more likely to result in a statistically significant difference.

Further, it is an invalid inference to conclude that a magnocellular deficiency exists solely based on the finding that a magnocellular condition gives statistically significant differences whereas a parvocellular condition does not. To do so would require making an interpretation of the non-significant data—but a statistically non-significant result does not allow any conclusions to be inferred (Gill, 1999). The finding that the difference is statistically significant under the magnocellular condition and not under the parvocellular condition, moreover, is not itself strictly pertinent. What might have been somewhat more relevant would have been if the difference between the data obtained under the two conditions were statistically significant. (It should in this connection be kept in mind that the difference between a statistically significant result and a non-significant result need not itself be statistically significant. Gelman and Stern, 2006). However, it is not clear that even this would have solved the problem since it may be possible for a single factor to have effects that are different under different conditions (as shown in Figure 1). The difference between these effects may, or may not, be statistically significant.

## Summary

We have demonstrated that the VEP data of Butler et al. (2009) are very well described by a general response reduction. This argues that abnormal visual performance in schizophrenia reflects a general reduction in sensitivity or response amplitude rather than a magnocellular deficiency.
